# Jamie's Ministry of Food: Quasi-Experimental Evaluation of Immediate and Sustained Impacts of a Cooking Skills Program in Australia

**DOI:** 10.1371/journal.pone.0114673

**Published:** 2014-12-16

**Authors:** Anna Flego, Jessica Herbert, Elizabeth Waters, Lisa Gibbs, Boyd Swinburn, John Reynolds, Marj Moodie

**Affiliations:** 1 Deakin Health Economics, Faculty of Health, Deakin University, Melbourne, Australia; 2 Jack Brockhoff Child Health and Wellbeing Program, Melbourne School of Population and Global Health, The University of Melbourne, Melbourne, Australia; 3 World Health Organisation Collaborating Centre for Obesity Prevention, Faculty of Health, Deakin University, Melbourne, Australia; 4 School of Population Health, Faculty of Medical and Health Sciences, University of Auckland, Auckland, New Zealand; 5 Biostatistics Unit, Faculty of Health, Deakin University, Melbourne, Australia; The Australian National University, Australia

## Abstract

**Objective:**

To evaluate the immediate and sustained effectiveness of the first Jamie's Ministry of Food Program in Australia on individuals' cooking confidence and positive cooking/eating behaviours.

**Methods:**

A quasi- experimental repeated measures design was used incorporating a wait-list control group. A questionnaire was developed and administered at baseline (T1), immediately post program (T2) and 6 months post completion (T3) for participants allocated to the intervention group, while wait -list controls completed it 10 weeks prior to program commencement (T1) and just before program commencement (T2). The questionnaire measured: participants' confidence to cook, the frequency of cooking from basic ingredients, and consumption of vegetables, vegetables with the main meal, fruit, ready-made meals and takeaway. Analysis used a linear mixed model approach for repeated measures using all available data to determine mean differences within and between groups over time.

**Subjects:**

All adult participants (≥18 years) who registered and subsequently participated in the program in Ipswich, Queensland, between late November 2011- December 2013, were invited to participate.

**Results:**

In the intervention group: 694 completed T1, 383 completed T1 and T2 and 214 completed T1, T2 and T3 assessments. In the wait-list group: 237 completed T1 and 149 completed T1 and T2 assessments. Statistically significant increases within the intervention group (P<0.001) and significant group*time interaction effects (P<0.001) were found in all cooking confidence measures between T1 and T2 as well as cooking from basic ingredients, frequency of eating vegetables with the main meal and daily vegetable intake (0.52 serves/day increase). Statistically significant increases at T2 were sustained at 6 months post program in the intervention group.

**Conclusions:**

Jamie's Ministry of Food Program, Australia improved individuals' cooking confidence and cooking/eating behaviours contributing to a healthier diet and is a promising community-based strategy to influence diet quality.

## Introduction

Healthy food choices and eating patterns are essential for promoting good health and well-being and preventing a wide range of chronic diseases [1] such as type two diabetes [2], cardiovascular disease(CVD) [3], stroke [4]some cancers [5], [6] and obesity [7], [8]. Yet it is increasingly more difficult for westernized societies to achieve population adherence to recommended nutrition guidelines for healthy eating [9]. As such, nutrition-related chronic disease and obesity is on the rise [10], and, in addition to placing considerable burden on healthcare budgets, is also having far wider impacts within societies [11], [12]. Poor diet has been identified as a leading contributing risk factor for global disease burden, with obesity, in part a product of unhealthy eating, being the leading risk factor in Australasia [13].

One concerning trend is the increasing consumption of energy dense foods of lower nutritional value prepared outside of the home [14] rather than the preparation and consumption of home cooked meals that are associated with higher vegetable intake [15] and overall higher diet quality [16]. This shift towards outsourcing of meal preparation [14] is likely to be the product of a multitude of varying, competing and inter-related factors [17], [18] including social, economic, cultural, environmental and technological influences, that serve to potentially constrain home cooking practices [14]. One hypothesis is that a decline in cooking skills in adults is contributing to the problem [19]. Caraher and Lang [19], in their review of the state of cooking skills in England, identified many possible reasons for declining cooking skills including a reduction in opportunities to learn to cook. In Australia, cooking is not always taught in schools and fundamental changes in the ways in which individuals and families function, on a day-to-day basis, in a modern society have reduced the traditional opportunities for learning to cook from family members. Furthermore there is growing evidence of a relationship between a lack of cooking skills, low cooking confidence and poor food choices [20], including the likelihood of higher ready prepared meal consumption [21]; inversely, higher levels of cooking skills and confidence are associated with higher vegetable purchasing [22] and healthier eating [15].

This evidence underpins the proliferation of community-based cooking skills education programs which are being used as a strategy, both by government and non -government organisations, to promote cooking skills and cooking confidence as a vehicle to healthy eating. For example, the recent World Cancer Research Fund's (WCRF) “NOURISHING” food policy framework specifically identifies cooking skills education within its nutrition education policy area, which is one of the ten key policy areas of action to foster healthy eating and prevent obesity [23]. The Cooking Matters program in the United States (US) and the Jamie Ministry of Food programs in the United Kingdom (UK) and now Australia are recognisable community-based cooking skills programs. The latter, in particular, has attracted attention, in part due to their development by Jamie Oliver, a UK based celebrity chef. However, the evidence to support the efficacy of these and other community-based cooking programs, in adult populations, is still emerging [24], [25]; the Jamie Ministry of Food program itself is yet to be formally evaluated. Individual programs have reported positive findings in relation to improved cooking confidence [26], [27], healthy cooking and food consumption patterns [27], [28], [29] with some sustained effects up to one year post intervention [27]. A recent systematic review concluded that interventions involving home food preparation and/or cooking may result in improved food choices, dietary behaviours and other health related outcomes [25]. Still the authors were cautious in their interpretation of the results given the dearth of suitably robust evaluations from which to make definitive statements about program effectiveness [25]. Given growing pressure for evidence-based public health investment [30], evaluations of program effectiveness should be both robust and practicable.

This study aims to evaluate the immediate impacts and longer term outcomes of Jamie's Ministry of Food program, in Ipswich, Australia using a quasi-experimental, repeated measures design. This paper reports the impacts of the program in terms of cooking confidence and cooking and eating behaviours. This quantitative component is embedded within a larger mixed methods evaluation [31]; other secondary outcomes (including a range of attitudinal, knowledge, affordability metrics and broader psychosocial impacts) and qualitative study findings are reported elsewhere [32].

## Methods

### The intervention

Jamie's Ministry of Food programs have been running since 2008 in the UK funded by a mixture of private and public sources. In 2010, Jamie's Ministry of Food was brought to Australia by The Good Foundation (TGF), a not-for-profit, health promotion organisation funded by The Good Guys, a major Australian electrical goods retailer, in partnership with Jamie Oliver. In April 2011, the first Australian Jamie's Ministry of Food Centre opened in Ipswich, Queensland (QLD). The Ipswich Centre is predominantly funded by philanthropist Mr Andrew Muir (owner of The Good Guys) and QLD State Government, as well as local partners. Whilst the program is all inclusive and not targeted towards a specific demographic group, Ipswich was deliberately chosen as the location due to its population's lower socioeconomic status [33] and higher prevalence of overweight and obesity than the QLD state average [34]. Given the socioeconomic gradient apparent in obesity prevalence within Australia [35], wherein health inequalities related to obesity and its risk factors such as unhealthy eating [36] are more evident in lower income groups, Ipswich was considered a suitable setting for the program.

Jamie Oliver's manifesto is to inspire individuals to cook simple basic meals from scratch both for themselves and their families [37].The 10 week program, consisting of weekly 1.5 hour classes, runs from a fixed site located in the main Ipswich shopping precinct. Participants are taught recipes from 50 “Jamie Oliver” recipes which have been adapted to the Australian context from the UK program so that over the 10 week course they learn to prepare and cook a variety of dishes while learning specific cooking techniques such as chopping, frying, roasting and baking. Messages about good nutrition, meal planning and budgeting are embedded in the program and are discussed in an informal manner during the skills sessions. There is an emphasis on cooking from scratch using fresh ingredients such as fish, meat and seasonal vegetables and fruit. Further details of the program, its specific objectives, and the full evaluation protocol are reported elsewhere [31].

### Evaluation Objectives

To provide a basis for evaluation enquiry, a program logic model was developed to determine the potential pathways to behaviour change [31]. Whilst some pathways such as the effect of cooking skills programs on individuals' cooking confidence/self -efficacy and cooking and eating behaviours were based on an emerging evidence base, and were directly aligned with program objectives, others around psychosocial, social connectedness, attitudinal and other broader impacts were viewed as more exploratory [31]. This paper draws on the quantitative evaluation to explore the former pathways and determine the impact of Jamie's Ministry of Food on participants' cooking confidence (self-efficacy), and cooking and eating behaviours.

### Evaluation design

The evaluation used a quasi-experimental design with a wait-list control group. All program participants aged 18 years and over were invited to participate in the evaluation. Participants who signed up to the program >10 weeks before their course start date, due to the length of the wait-list at the time, were allocated to the control group whilst participants who signed up to the program <10 weeks before their course start date, were allocated to the intervention group. Intervention participants were surveyed at three time points (before the program start (T1), on program completion (T2) and six months after program completion (T3), whilst control participants were surveyed 10 weeks before program start date (T1) and just prior to beginning their first class (T2). An online questionnaire was used followed by a postal version sent to all non-respondents or to anyone who did not have access to a computer or the internet. Data collection ran from December 2011 to December 2013. Six month follow up data were not collected from controls; instead, data from Queensland State-wide monitoring of vegetable intake [38] collected and reported during the evaluation data collection period was used as a proxy T3 control measurement to enable comparison with the self- reported vegetable intake measured in the intervention group at 6 months post intervention (T3). Further details of the methods are provided in Flego et al 2013 [31]. The evaluation was approved by the Deakin University Human Research Ethics Committee (HEAG-H 117_11) in October 2011 and was registered with the Australian and New Zealand Clinical Trials Registry (registration number: ACTRN12611001209987).

### Questionnaire and outcome measures

A self -administered quantitative measurement tool was developed [31]. The primary outcome measures for the quantitative evaluation were a change in self-reported cooking confidence and daily vegetable intake. Cooking confidence (self-efficacy) was measured by five questions assessing confidence in a number of generalised skills required to do basic cooking at home on a scale from 1-5 anchored from “not at all confident” = 1 to “extremely confident”  = 5. Four of the confidence questions were adapted from a validated cooking skills questionnaire by Barton et al, 2011 [39] and the fifth adapted from Keller et al, 2004 [40]. In addition to the separate confidence items, all item scores were combined to create an overall confidence score. Self-reported daily vegetable intake was measured as serves per day (a serve was equal to half a cup of cooked vegetables or 1 cup of salad where cup  = 250 ml) anchored from 0 =  no serves per day to 6 or more serves per day  = 6. This question used the same wording as that used in the Queensland Self-Reported Health Status Survey question to enable comparison [41].

Secondary outcomes included changes in self-reported cooking and eating measures including: (i) frequency of cooking the main meal from basic ingredients; (ii) frequency of eating ready-made meals at home; (ii) frequency of eating vegetables with the main meal, all measured on a scale from 0 =  never to 7 =  daily; (iv)serves of fruit per day measured as per daily vegetable intake and (v) frequency of eating takeaway per week measured from 0 =  never to 5 = 5 or more times per week.

### Sample size

Sample size calculations were based on the primary outcome of a change in self-reported vegetable intake. The study was powered to detect a mean change in self-reported vegetable intake of 0.5 serves per day, at 80% power (0.05 significance) starting from a baseline measure of 2.5 serves a day [41]. This assumed the use of a nested anova or mixed model analysis with an F test for the group by time interaction. Additional sample size calculations suggested at least 140 participants would be required in each group to detect a mean difference of 0.5–0.7 serves per day. For further details of sample size calculations, see Flego et al, 2013 [31].

### Statistical Analysis

All continuous outcomes were analysed using linear mixed models for repeated measures to determine mean differences between groups over time. This type of analysis copes with unbalanced groups, missing (at random) follow up data and enables all available data to be utilised to determine the estimated changes in mean outcomes. Predicted means from the mixed model analyses are reported in all tables in the results section. Each analysis was also subsequently run with separate adjustments for gender, age (dichotomised at below 50 or 50 years and above) and categories of employment status then with all three covariates together, to account for the potential effect of baseline differences (assessed by chi-squared tests between groups, refer to Table 1) on the estimated effect of the intervention.

**Table 1:** Demographic characteristics of evaluation participants by time point a.

Group, Time-point	Intervention T1	Intervention T2	Intervention T3	Control T1	Control T2
	% (n)			% (n)	
**Gender** b					
Female	77.4 (525)	79.1(299)	80.5(207)	87.2 (198)	87.7(128)
Male	22.6 (153)	20.9(79)	19.5(50)	12.8 (29)	12.3(18)
**Age (years)**					
Under 50^b^	55.6 (375)	44.1(165)	43.5(110)	64.3 (144)	60.3(85)
50 and over b	44.4 (300)	55.9 (209)	56.5(143)	35.7 (80)	39.7(56)
18–24	7.4 (50)	2.7(10)	3.2(8)	5.8 (13)	4.3(6)
25–34	17.5 (118)	14.2(53)	14.6(37)	22.8 (51)	20.6(29)
35–44	23.0 (155)	19.8(74)	19.0(48)	26.3 (59)	24.1(34)
45–54	16.0 (108)	15.5(58)	15.8(40)	16.5 (37)	18.4(26)
55–64	15.0 (101)	18.45(69)	18.6(47)	12.5 (28)	13.5(19)
65–74	17.5 (118)	24.6(92)	24.1(61)	13.4 (30)	15.6(22)
75+	3.70 (25)	4.8(18)	4.7(12)	2.70 (6)	3.5(5)
Mean age years (SD)	48(16.1)	52(15.7)	52(15.9)	46(15.1)	47(15.2)
**Aboriginal and/or Torre Strait Islander**	1.8 (12)	1.8(7)	2.7(7)	0.9 (2)	1.4(2)
**Speaks a language other than English at home**	7.8 (53)	6.9(26)	7.4(19)	5.3 (12)	3.4(5)
**Locality**					
Ipswich	82.0 (555)	84.7(320)	83.3(214)	78.8 (178)	79.4(116)
Other Queensland localities	17.7 (120)	15.3 (58)	16.3(42)	21.2 (48)	20.5 (30)
NSW	0.3 (2)	0.0(0)	.4(1)	0.0 (0)	0.0
**Highest Level of education attained**					
High school, year 12 or less	47.8 (321)	49.3(185)	49.4(126)	45.8 (104)	47.3(69)
TAFE, apprenticeship, diploma or certificate	22.2 (149)	20.8(78)	21.6(55)	22.9 (52)	19.9(29)
Tertiary, bachelor degree or higher	28.0 (188)	28.5(107)	27.4(70)	29.1 (66)	30.1(44)
Other	2.0 (13)	1.3(5)	1.6(4)	2.2 (5)	2.7(4)
**Employment** b					
Full-time	26.4 (176)	23.6(88)	26.2(67)	34.7 (79)	31.3(46)
Part-time/casual	18.6 (124)	16.6(62)	18.4(47)	14.5 (33)	17.0(25)
Retired	23.8 (159)	31.6(118)	30.5(78)	21.5 (49)	23.8(35)
Home duties/carer	14.4 (96)	15.3(57)	13.3(34)	18.4 (42)	17.7(26)
Not working (Permanently ill/unable to work, unemployed)	9.9 (66)	7.0(26)	4.3(11)	8.8 (20)	7.5(11)
Student (full-time and part-time)	3.1 (21)	1.9(7)	1.9(5)	1.3 (3)	2.0(3)
Other	3.9 (26)	4.0(15)	5.47(14)	0.9 (2)	.70(1)
**Household yearly income**					
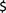 1– 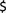 6,000	2.5(15)	1.8(6)	2.6(6)	2.0(4)	1.5(2)
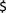 6,001– 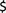 13,000	5.7(34)	5.7(19)	5.3(12)	5.0(10)	5.3(7)
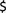 13,001– 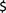 20,000	11.9(71)	12.9(43)	14.1(32)	9.5(19)	9.9(13)
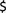 20,001– 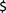 30,000	14.8(88)	17.7(59)	17.2(39)	9.5(19)	9.9(13)
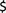 30,001– 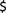 50,000	15.4(92)	14.7(49)	14.5(33)	12.5(25)	12.2(16)
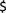 50,001– 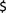 100,000	30.0(179)	29.7(99)	26.9(61)	35.5(71)	36.6(48)
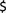 100,001– 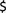 150,000	13.6(81)	11.1(37)	12.3(28)	18.5(37)	16.8(22)
>150,000	6.0(36)	6.3(21)	7.0(16)	7.5(15)	7.6(10)
**Household Characteristics**					
Couple, with young children (0–17 years old) living at home	24.7 (169)	23.2(88)	20.0(51)	32.1 (76)	30.2(45)
Couple, with adult children (18 years and over) living at home	12.5 (86)	10.8(41)	11.0(28)	10.1 (24)	10.7(16)
Couple, without children living at home	32.9 (226)	35.3(134)	36.0(92)	24.5 (58)	27.5(41)
One parent family with children living at home	7.0 (48)	3.7(14)	4.3(11)	8.9 (21)	6.7(10)
Live Alone	16.0 (110)	21.6(82)	22.7(58)	17.7 (42)	20.1(30)
Other	6.9 (47)	5.5(21)	6.2(16)	6.8 (16)	4.7(7)
**Mean household size (SD)** c	2.8 (1.5)	2.6(1.3)	2.5(1.3)	3.0(1.6)	2.9(1.6)
**Median household size** c **(50%centile)**	2	2	2	3	2

a Sample size for different variables might vary from total sample size because of missing responses and rounding of weighted frequencies.

b significant difference between groups (p<0.05) at baseline as tested with chi squared analysis.

c excludes 2 participants living in institutional facilities.

SD  =  standard deviation.

Ratings on the cooking confidence score from “not at all confident” (1) to “extremely confident” (5) were regarded as a continuous variable in the primary analysis - an acceptable technique for larger data sets [42]. However, supplementary analyses using ordinal logistic and logistic models, to account for the ordered response categories, and the dichotomization of the categories (“not confident” and “confident”), were also conducted to determine if results from the differing statistical approaches would provide similar inferences with respect to differences between groups in their changes over time (see S3 and S4 Tables).

To explore the sustainability of the intervention group effect over time, a repeated measures analysis was performed using the intervention group data collected at all three time points. For the primary outcome measure of self-reported vegetable intake, a two-sample t-test comparison between the 6-month post-program intervention group mean and the reported state-wide mean was performed.

All analyses were performed using STATA software (version 12.0). Results were deemed significant at the P<0.05 level.

## Results

### Participant recruitment and retention

**Figure 1:**
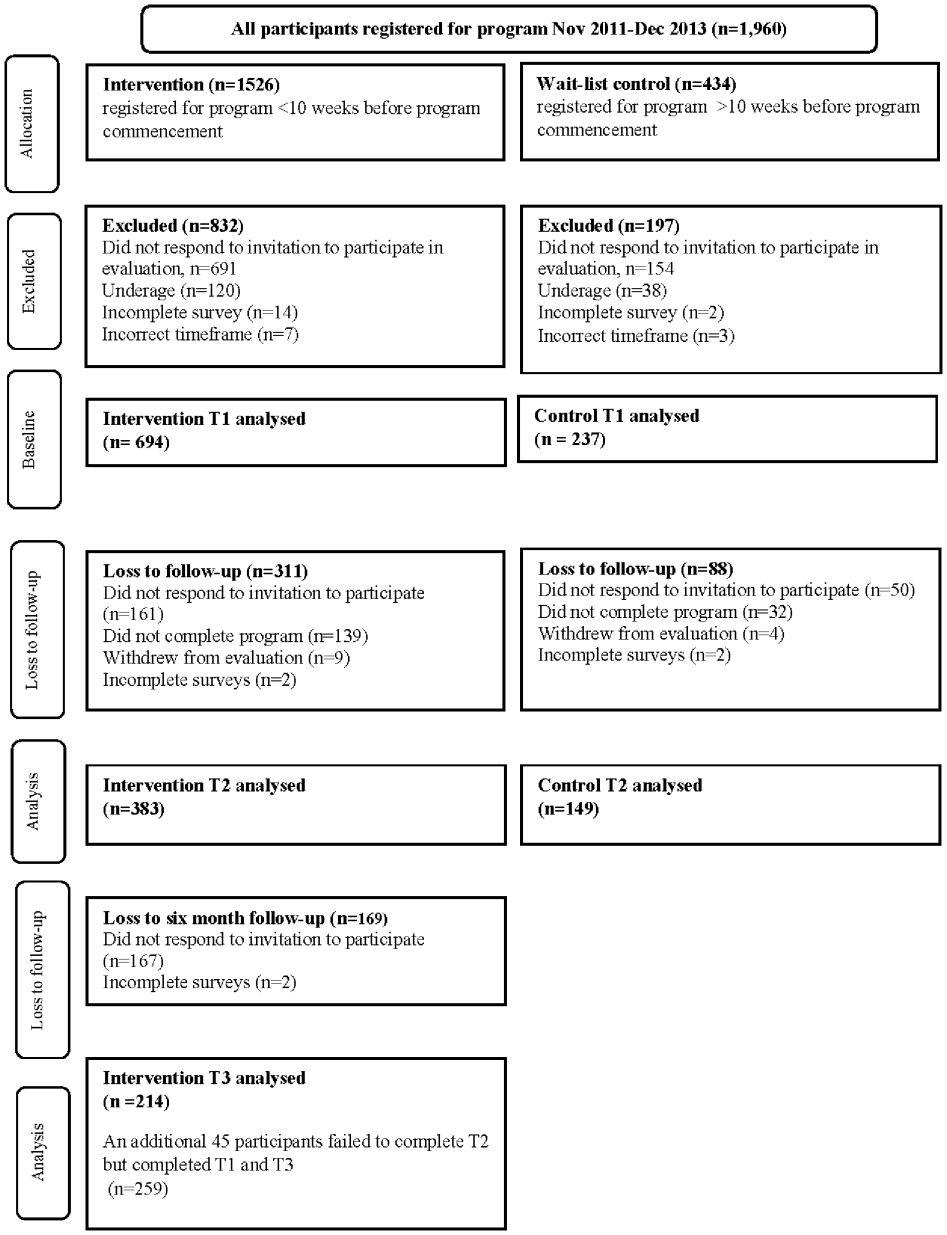
Evaluation participation over time.

Over the two-year data collection period, approximately 1960 participants registered for the program and were invited to participate in the evaluation; 1526 were allocated to the intervention group and 434 to the control group. Fig. 1 provides a summary of group allocation and response numbers including specification of exclusions and loss to follow up at each time point.

Some 45% of intervention and 55% of control participants invited to participate in the evaluation completed the baseline assessment (T1). Of those participants who completed baseline, 55% of intervention and 63% of control participants completed the T2 assessment. In the intervention group, 31% of those participants who completed baseline, also completed both T2 and T3 assessments.

### Baseline demographic profile

At baseline (Table 1), the majority of participants (∼80%) were female, with a statistically significant higher proportion of females in the control group (87.2%) than the intervention group (77.4%) (P<0.05). Both groups had more participants aged under 50 years than over, with the controls having significantly more in the younger category than the intervention group (64% and 55% respectively P<0.05). Employment status differed between groups (P<0.05) with the intervention group less likely to be in full time employment (26%) and more likely to be retired (24%) compared to the control group (35% and 21% respectively). Losses to follow-up in each group over time significantly differed by age (P<0.05) but not for gender (P>0.05) (Table 1).

### Change in cooking confidence (self-efficacy)

In the intervention group but not the control group, there was a statistically significant increase between T1 and T2 in all cooking confidence measures (P<0.001) both individually and when combined into a cooking confidence score (Table 2). Statistically significant group by time interactions for all confidence measures (P<0.001) demonstrate that the change over time differed between groups. These results remained significant at the P<0.001 level for all covariate-adjusted analyses performed for each confidence variable (see S1 Table). The supplementary ordinal logistic and logistic analyses (S3 and S4 Tables) also resulted in similar findings regarding the differences between the groups for all cooking confidence measures (P<0.001).

**Table 2:** Cooking and eating measures at baseline and immediately post program^1^.

	Intervention group			Control group			Difference between groups in changes from baseline (interaction effect)^3^ P value
Outcome measure	Baseline value(T1) mean (S.E)^2^	Follow up value(T2) mean (S.E)	Change from baseline(T2-T1) mean (S.E) P value	Baseline value(T1) mean (S.E)^2^	Follow up value(T2) mean (S.E)	Change from baseline(T2-T1) mean (S.E) P value	*P value*
***Cooking confidence***							
Confidence to cook from basic ingredients^4^	3.56(0.04)	4.36(0.05)	0.81(0.05) p<0.001	3.69(0.07)	3.72(0.08)	0.03(0.08) p = 0.70	p<0.001
Confidence to follow a simple recipe^4^	4.00(0.04)	4.53(0.05)	0.53(0.04) p<0.001	4.11(0.06)	4.06(0.07)	−0.06(0.07) p = 0.40	p<0.001
Confidence in preparing and cooking new foods and recipes^4^	3.35(0.04)	4.13(0.05)	0.77(0.05) p<0.001	3.45(0.07)	3.55 (0.08)	0.10(0.08) p = 0.22	p<0.001
Confidence that what one cooks will turn out well^4^	3.21(0.04)	3.93(0.05)	0.72(0.04) p<0.001	3.28(0.06)	3.35(0.07)	0.07(0.07) p = 0.30	p<0.001
Confidence to taste new foods never eaten before^4^	3.47(0.04)	4.01(0.05)	0.54(0.05) p<0.001	3.41(0.07)	3.51(0.09)	0.09(0.08) p = 0.25	p<0.001
Combined confidence score^5^	17.6(0.02)	21.0(0.2)	3.36(0.18) p<0.001	17.9(0.03)	18.2(0.03)	0.23(0.28) p = 0.41	p<0.001
***Cooking and eating measures***							
Cooking the main meal from basic ingredients^6^	4.05 (0.08)	4.66 (0.09)	0.61 (0.09) p<0.001	4.16 (0.14)	4.17(0.16)	0.01(0.15) p = 0.95	p<0.001
Consumption of ready- made meals at home^6^	1.06 (0.05)	0.95 (0.06)	−0.11 (0.06)p = 0.06	1.11(0.08)	1.21(0.10)	0.10(0.10) p = 0.30	p = 0.06
Consumption of vegetables with the main meal^6^	4.67(0.07)	5.06(0.09)	0.39 (0.08) p<0.001	4.76(0.12)	4.75(0.14)	0.01(0.14) p = 0.94	p = 0.01
Daily vegetable consumption (serves per day)	2.46 (0.51)	2.97(0.06)	0.52 (0.06) p<0.001	2.49(0.09)	2.59(0.10)	0.10(0.10) p = 0.30	p<0.001
Daily fruit consumption (serves per day)	1.65(0.04)	1.93(0.05)	0.28 (0.05) p<0.001	1.61(0.07)	1.71(0.08)	0.10(0.08) p = 0.20	p = 0.06
Take-away consumption^6^	0.98(0.04)	0.77(0.04)	−0.21(0.04) p<0.001	0.94(0.06)	0.96(0.07)	0.03(0.06) p = 0.62	p = 0.001

1 Outcomes within each group and over time were determined by a mixed linear model for repeated measures using all available data at each time point. All means and Standard Errors (S.E) have been rounded to 2 decimal points.

2 Baseline values were not significantly different between groups (independent t tests P<0.05).

3 A significant group x time interaction effect denotes that the response over time differed between groups.

4 Scale values are 1–5 (where 1 =  not at all confident and 5 =  extremely confident and 4 or>  =  confident).

5 The combined confidence score is equal to the sum total of all other confidence scores (scores 20 or>  =  confident).

6 Times per week.

When analysis was restricted to the intervention group only to test the sustained effect of the program (i.e. to T3), statistically significant increases in all cooking confidence measures (P<0.001) were reported for pairwise comparisons between T1 and T2 (P<0.001) and T1 and T3 (6 months post intervention) (P<0.001) but not between T2 and T3 (Table 3). Statistically significant results were also noted (P<0.001) for the overall or main effects of time.

**Table 3:** Cooking and eating measures for the intervention group only at baseline, post intervention and 6 months follow up^1^.

	intervention group						overall effect of change over time P value
Outcome measure	Baseline value(T1) mean (S.E)	Follow up value(T2) mean (S.E)	6 months post intervention follow up (T3) mean (S.E)	Change from baseline(T2-T1) mean (S.E) P value	Change from baseline(T3-T1) mean (S.E) P value	Change between follow up (T3-T2) mean (S.E) P value	*P value*
***Cooking confidence***							
Confidence to cook from basic ingredients^2^	3.56(0.04)	4.37(0.05)	4.43(0.06)	0.81(0.05)p<0.001	0.87(0.06)p<0.001	0.07(0.06)p = 0.280	p<0.001
Confidence to follow a simple recipe^2^	4.00(0.04)	4.53(0.04)	4.61(0.05)	0.53(0.04)p<0.001	0.61(0.05)p<0.001	0.08(0.05)p = 0.133	p<0.001
Confidence in preparing and cooking new foods and recipes^2^	3.35(0.04)	4.13(0.05)	4.17(0.06)	0.78(0.05)p<0.001	0.82(0.06)p<0.001	0.05(0.06)p = 0.439	p<0.001
Confidence that what one cooks will turn out well^2^	3.21(0.04)	3.93(0.05)	3.94(0.05)	0.72(0.04)p<0.001	0.73(0.05)p<0.001	0.01(0.06)p = 0.803	p<0.001
Confidence to taste new foods never eaten before^2^	3.47(0.04)	4.01(0.05)	3.99(0.06)	0.53(0.05)p<0.001	0.52(0.06)p<0.001	−0.02(0.06)p = 0.746	p<0.001
Combined confidence score^3^	17.59(0.16)	20.95(0.19)	21.15(0.22)	3.36(0.18)p<0.001	3.56(0.21)p<0.001	0.20(0.22)p = 0.363	p<0.001
***Cooking and eating measures***							
Cooking the main meal from basic ingredients^4^	4.05(0.08)	4.65(0.10)	4.88(0.11)	0.60(0.09) p<0.001	0.84 (0.10) p<0.001	0.24(0.11) p = 0.03	p<0.001
Consumption of ready- made meals at home^4^	1.06(0.05)	0.93(0.06)	0.80(0.07)	−0.13(0.06)p = 0.04	−0.26(0.07)p<0.001	−0.13(0.08) p = 0.09	p<0.001
Consumption of vegetables with the main meal^4^	4.67(0.07)	5.05(0.09)	5.31(0.10)	0.38(0.09) p<0.001	0.64(0.09) p<0.001	0.25(0.10) p<0.018	p<0.001
Daily vegetable consumption (serves per day)	2.46(0.05)	2.97(0.06)	3.05(0.07)	0.51(0.06)p<0.001	0.60(0.07)p<0.001	0.08(0.08)p = 0.273	p<0.001
Daily fruit consumption (serves per day)	1.65(0.04)	1.93(0.05)	2.05(0.06)	0.27(0.05) p<0.001	0.40(0.06) p<0.001	0.12(0.06) p = 0.055	p<0.001
Take-away consumption^4^	0.98(0.04)	0.76(0.04)	0.73(0.05)	−0.23(0.04)p<0.001	−0.25(0.04)p<0.001	−0.02(0.05)p = 0.607	p<0.001

1 Outcomes at each time point were determined by a mixed linear model for repeated measures using all available data at each time point. All means and Standard Errors (S.E) have been rounded to 2 decimal points.

2 Scale values are 1–5 (where 1 =  not at all confident and 5 =  extremely confident).

3 The combined confidence score is equal to the sum total of all other confidence scores (scores 20 or>  =  confident).

4 Times per week.

### Self -reported Vegetable intake

Self-reported daily vegetable intake increased significantly between T1 and T2 in the intervention group by just over a half serve (0.52 serves, SD 0.06, P<0.001) but not in the control group (0.10 serves, SD 0.1, P = 0.30). A statistically significant group by time interaction (P<0.001) was found (Table 2). All adjusted analyses found very similar results (see S2 Table).

For the intervention group only, daily vegetable consumption increased significantly from T1 (2.46, SD 0.05) to T2 (2.97, SD 0.06, P<0.001) and T3 (3.05, SD 0.07), although the change between T2 and T3 was not significant (P = 0.273). The change between T1 and T3 was significant (0.60 serves, SD 0.07, P<0.001) as was the overall effect of change over time (P<0.001) (Table 3).

Results of the t-test comparison between the self-reported mean vegetable intake at T3 for the intervention group (mean = 3.13, SD = 1.39) and state-wide monitoring data from the 2012 Queensland Self-Reported Health Status Survey (mean = 2.39, SD  =  2.39) [38] showed a statistically significant mean difference of 0.74 serves (SD 0.09, P<0.001).

### Changes in other cooking and eating measures

Cooking the main meal from basic ingredients increased significantly from T1 to T2 in the intervention group (0.61, SD 0.09, P<0.001) and the overall group by time interaction effect was statistically significant (P<0.001) (Table 2). Both the consumption of takeaway food and of ready -made meals reduced in the intervention group although only the reduction in takeaway consumption was statistically significant (-0.21, SD 0.04, P<0.001) and showed a significant interaction effect (P<0.001). Consumption of vegetables with the main meal and daily fruit intake also increased significantly in the intervention group (P<0.001) but daily fruit intake did not demonstrate a significant group by time interaction effect (P = 0.06). No significant differences in any of the cooking and eating measures were found in the control group between T1 and T2. All adjusted analyses performed found very similar results (see S2 Table).

Analysis of data from the intervention group only (Table 3) showed an overall statistically significant positive effect over time for the aforementioned cooking and eating measures (P<0.001) and between T1 and T2 (P<0.05), however change between T2 and T3 did not remain significant for reductions in consumption of ready-made meals or takeaway, or for increases in daily fruit intake.

## Discussion

This is the first published quantitative evaluation of Jamie's Ministry of Food Program providing evidence of the program's impact on participants' cooking and eating behaviours. While evaluation recruitment was relatively modest at baseline (∼47% response rate), the retention rates were similar to other recent studies in this area [27]. Results showed that the program significantly increased participants' cooking confidence in all generalised cooking skill areas tested from baseline (T1) to program completion (T2) and the increase in the intervention group was also sustained six months later which is indicative of an enduring program effect. These findings resonate with those from Garcia et al, 2012 who also found sustained positive effects of a cooking skills program on participants' cooking confidence at one year post intervention [27]. Statistically significant increases between T1 and T2 were also found in the weekly frequency of cooking the main meal from basic ingredients and in the consumption of vegetables with the main meal together with a reduction in weekly take-away consumption, although effect sizes were modest. Whilst daily fruit consumption increased in the intervention group between baseline and both T2 and T3, there was no significant difference between groups at T2. Daily vegetable intake however increased significantly by just over a half a serve per day within the intervention group from baseline to T2, but not in the control group, and continued to increase (but not significantly), between T2 to T3. The T3 intervention mean vegetable intake was significantly higher, by 0.74 serves per day, than the reported Queensland State-wide monitoring result [38].

These findings are encouraging and align with evaluation of other cooking skills programs that have found positive results with respect to improved cooking confidence [26], [27] and/or healthy eating results [25]. In particular, the 0.5 serve increase in vegetable intake, which was maintained at 6 months follow up in the intervention group, is comparable to outcomes achieved by other nutrition education programs exclusively targeting healthy eating in community settings [43] or low income communities [43]. In light of published relative risk reductions for coronary heart disease of 7% associated with a one serve per day increase in vegetable consumption [44] as well as reported risk reductions for stroke [45] with increased vegetable consumption, an increase of 0.5 serves of vegetables is likely to have small but positive protective benefits for individuals and arguably be of public health benefit if achieved across whole populations. It has been estimated that inadequate fruit and vegetable consumption alone, cost the Australian health sector AUD 206 million in 2008 [46]; therefore small shifts in the right direction are a constructive first step.

Additionally, the apparent agreement between results around daily vegetable intake, consumption of vegetables with the main meal, cooking confidence and cooking from basic ingredients, all of which reported statistically significant increases, suggests that the program influences many aspects of cooking and eating behaviours simultaneously and the parallel increases in these variables are consistent with the causal logic model proposed for the program [31]. The results of the intervention only analysis over time also implies a sustaining of program effect 6 months post program for most variables reported. However, there was very little change from completion (T2) to 6 months post program (T3), therefore in agreement with Garcia et al [27], a refresher or booster class around this time may be warranted to enhance program effect in the long run.

Furthermore, acknowledging that self-reported daily fruit intake did not result in a statistically significant difference between groups at T2 (P = 0.06), a 0.40 serves per day increase was still found between T1 and T3 in the intervention group only analysis (P<0.001). Nevertheless the statistically significant results between groups for self-reported vegetable intake and not for self-reported fruit intake possibly reflect the emphasis within the Jamie's Ministry of Food program to promote and teach inclusion of vegetables or salad with the main meal.

The results of this study offer the strongest quantitative evidence at this point in time to specifically support the premise that Jamie's Ministry Food program improves cooking confidence and leads to healthier cooking and eating behaviours. The evaluation also adds to the literature more generally around the effectiveness of cooking skills programs per se, particularly given the relatively large sample size, use of a control group and six month follow up measures, all design elements which have been lacking in many other studies of community cooking skills interventions [24]. It is acknowledged that there is a reliance on self-reported data which is not as valid as other more objective measurement tools used in traditional nutrition evaluation [47]. Yet the self-administered questionnaire, with a focus on simple English, was well completed, which was an important consideration, given the program was based in an area of social disadvantage. The use of objective measurement tools would have been difficult in this context because of the participant burden, as shown in previous studies [26]. Efforts were also made to increase data validity: guidance was provided to respondents on portion size, plus a question was included on vegetable intake within the main meal which is considered good practice [48]; also, importantly, questions used in Government preventive health surveillance surveys were included for comparison. As an aside, baseline mean daily vegetable intake reported in both groups was similar (2.46, 2.49 for each group) to that reported by the Queensland surveillance survey (2.4) [38] suggesting that our sample was a valid representation of the population. A wait-list, non-randomised study design was selected as the most suitable for this context and ensured participants were able to participate with family and friends at a convenient time. This led to some disparity between groups in terms of numbers and demographic characteristics at baseline including gender, age and employment status. However by adjusting for these covariates, the sensitivity of the group comparison to between- group differences at baseline, was able to be tested and proven to have little impact on results. The inclusion of 6 month follow up results in this study demonstrates the sustainability of effects on intervention participants. While it would have been ideal to extend the control comparison to this point, it was not feasible to expect wait-list participants to wait a further 6 months before starting the program. It has been recommended that studies into community cooking skills interventions ideally use a community control group [24] to avoid this issue all together, but this was not considered a feasible option for this evaluation.

Jamie's Ministry of Food Program in Australia, has succeeded in recruiting large numbers in the communities in which it is operating and has attracted both private and public investment even when it precedes evidence of program effectiveness. This may speak to the influence of Jamie Oliver himself as program ambassador and his capacity to mobilise various players within communities. Whether or not this celebrity endorsement augments program effectiveness and what role such programs should play in the promotion of public health nutrition, remain questions for future research as does determination of the efficiency of the program measured against known alternatives through cost-effectiveness analysis. However the results suggest that the Jamie's Ministry of Food Program holds promise as a community-based strategy and should certainly be considered as part of a comprehensive approach to improving diet quality.

## Conclusion

This evaluation has, for the first time, demonstrated that the Jamie's Ministry of Food Program has positive personal, dietary and likely health impacts for participants through improvements in cooking confidence, cooking and eating behaviours towards a healthier diet. These benefits were sustained at 6 months post program suggesting the program does have an enduring effect on program participants and should be considered as a component in any suite of interventions targeting healthy eating.

## Supporting Information

S1 Table Cooking confidence measures at baseline and follow up adjusted by age, gender and levels of employment independently and all together.
(DOCX)

S2 Table Cooking and eating measures at baseline and follow up adjusted by age gender and levels of employment independently and all together.
(DOCX)

S3 Table Results of ordinal logistic regression for confidence to cook questions.
(DOCX)

S4 Table Results of the logistic models for dichotomised confidence questions (“not confident” and “confident”).
(DOCX)
