# Supplementary material for: Jamie's Ministry of Food: Quasi-Experimental Evaluation of Immediate and Sustained Impacts of a Cooking Skills Program in Australia
Source: PLoS One. 2014 Dec 16;9(12):e114673. doi: 10.1371/journal.pone.0114673 (PMC4267737; doi:10.1371/journal.pone.0114673)
Supplement: S1 Table — Cooking confidence measures at baseline and follow up adjusted by age, gender and levels of employment independently and all together. (DOCX) [file pone.0114673.s001.docx]

| **Table S1: Cooking confidence measures at baseline and follow up¹ adjusted by age^2^, gender and levels of employment^3^ independently and all together.** | | | | | | | | |  |
| --- | --- | --- | --- | --- | --- | --- | --- | --- | --- |
|  |  | |  |  | |  |  |  |  |
|  | **intervention group** | | | | | **wait list control group** | | | **Interaction effect^5^ P value** |
| **Outcome measure** | **baseline value(T1) mean (S.E)^4^** | | **follow up value(T2) mean (S.E)** | | **change from baseline(T2-T1) mean (S.E) P value** | **baseline value(T1) mean (S.E)^4^** | **follow up value(T2) mean (S.E)** | **change from baseline(T2-T1) mean (S.E) P value** | ***P value*** |
| ***Cooking confidence*** |  | |  | |  |  |  |  |  |
| **Confidence to cook from basic ingredients^6^** |  | |  | |  |  |  |  |  |
| Age | 3.55(0.04) | | 4.37(0.04) | | 0.81(0.04) P<0.001 | 3.72(0.08) | 3.76(0.07) | 0.04(0.08) P=0.609 | P<0.001 |
| Gender | 3.56(0.04) | | 4.38(0.04) | | 0.81(0.04) P<0.001 | 3.69(0.08) | 3.74(0.07) | 0.05(0.07) P=0.62 | P<0.001 |
| Employment | 3.57(0.04) | | 4.37(0.05) | | 0.80(0.05) P<0.001 | 3.70(0.07) | 3.73(0.08) | 0.03(0.08) P=0.712 | P<0.001 |
| Age, gender, employment | 3.58(0.04) | | 4.38(0.05) | | 0.80(0.05) P<0.001 | 3.70(0.07) | 3.73(0.08) | 0.04(0.08) P=0.48 | P<0.001 |
| **Confidence to follow a simple recipe^6^** |  | |  | |  |  |  |  |  |
| Age | 3.99(0.04) | | 4.53(0.04) | | 0.54(0.04) P<0.001 | 4.15(0.07) | 4.07(0.06) | -0.08(0.07) P=0.211 | P<0.001 |
| Gender | 4.00(0.04) | | 4.54(0.04) | | 0.53(0.04)P<0.001 | 4.12(0.07) | 4.07(0.06) | -0.05(0.07) P=0.482 | P<0.001 |
| Employment | 4.00(0.04) | | 4.53(0.04) | | 0.53(0.04)P<0.001 | 4.13(0.06) | 4.07(0.07) | -0.06(0.07) P=0.368 | P<0.001 |
| Age, gender, employment | 4.02(0.04) | | 4.55(0.04) | | 0.53(0.04) P<0.001 | 4.12(0.06) | 4.04(0.07) | -0.07(0.07) P=0.295 | P<0.001 |
| **Confidence in preparing and cooking new foods and recipes^6^** |  |  | | |  |  |  |  |  |
| Age | 3.35(0.04) | | 4.12(0.04) | | 0.77(0.05) P<0.001 | 3.45(0.07) | 3.55(0.07) | 0.10(0.07) P=1.33 | P<0.001 |
| Gender | 3.36(0.04) | | 4.13(0.04) | | 0.77(0.05) P<0.001 | 3.44(0.07) | 3.54(0.07) | 0.09(0.08) P=0.223 | P<0.001 |
| Employment | 3.36(0.04) | | 4.13(0.05) | | 0.77(0.05)P<0.001 | 3.44(0.07) | 3.54(0.08) | 0.01(0.08) p=0.212 | P<0.001 |
| Age, gender, employment | 3.37(0.04) | | 4.13(0.05) | | 0.77(0.05) P<0.001 | 3.44(0.07) | 3.54(0.08) | 0.01(0.08) P=0.224 | P<0.001 |
| **Confidence that what one cooks will turn out well^6^** |  | |  | |  |  |  |  |  |
| Age | 3.21(0.04) | | 3.93(0.04) | | 0.72(0.04) P<0.001 | 3.30(0.06) | 3.37(0.07) | 0.08(0.07) P=0.264 | P<0.001 |
| Gender | 3.21(0.04) | | 3.93(0.04) | | 0.72(0.04) P<0.001 | 3.30(0.06) | 3.36(0.07) | 0.06(0.07) P=0.353 | P<0.001 |
| Employment | 3.22(0.04) | | 3.93(0.05) | | 0.71(0.04) P<0.001 | 3.29(0.06) | 3.35(0.07) | 0.07(0.07) P=0.340 | P<0.001 |
| Age, gender, employment | 3.22(0.04) | | 3.93(0.05) | | 0.71(0.04) P<0.001 | 3.30(0.06) | 3.93(0.05) | 0.07(0.07) P=0.301 | P<0.001 |
| **Confidence to taste new foods never eaten before^6^** |  | |  | |  |  |  |  |  |
| Age | 3.46(0.04) | | 4.01(0.05) | | 0.55(0.05) P<0.001 | 3.43(0.08) | 3.52(0.08) | 0.09(0.08)P=0.266 | P<0.001 |
| Gender | 3.46(0.04) | | 4.00(0.05) | | 0.54(0.05) P<0.001 | 3.45(0.08) | 3.56(0.07) | 0.10(0.08) P=0.184 | P<0.001 |
| Employment | 3.47(0.04) | | 4.01(0.05) | | 0.54(0.05) P<0.001 | 3.44(0.07) | 3.52(0.09) | 0.10(0.08) P=0.219 | P<0.001 |
| Age, gender, employment | 3.46(0.04) | | 4.01(0.05) | | 0.55(0.05) P<0.001 | 3.44(0.08) | 3.55(0.09) | 0.10(0.08) P=0.212 | P<0.001 |
| **Combined confidence score^7^** |  | |  | |  |  |  |  |  |
| Age | 17.58(0.17) | | 20.98(0.17) | | 3.39(0.17) P<0.001 | 18.05(0.3) | 18.27(0.28) | 0.22(0.28) P=0.421 | P<0.001 |
| Gender | 17.60(0.17) | | 20.98(0.17) | | 3.39(0.17) P<0.001 | 18.01(0.30) | 18.27(0.28) | 0.26(0.27)P=0.344 | P<0.001 |
| Employment | 17.62(0.16) | | 20.98(0.20) | | 3.36(0.18) p<0.001 | 18.00(0.28) | 18.24(0.32) | 0.24(0.29) P=0.409 | P<0.001 |
| Age, gender, employment | 17.64(0.16) | | 21.01(0.20) | | 3.36(0.18) P<0.001 | 18.00(0.28) | 18.24(0.33) | 0.24(0.29) P=0.406 | P<0.001 |

¹Outcomes within each group and over time were determined by a mixed linear model for repeated measures using all available data at each time point. All means and standard errors (S.E) rounded to 2 decimal points

^2^ Age dichotomised at 50 years (below 50 years and 50 years and above)

^3^ Levels of employment status where 1= full time, 2= part time, 3= home duties/carer, 4= not working (permanently ill/unemployed), 5= student, 6= retired, 7=other

^4^Baseline values were not significantly different between groups (independent t tests)

^5^A significant group x time interaction effect denotes that the response over time differed between groups

^6^Scale values are 1-5 (where 1= not at all confident and 5 = extremely confident and 4 or > = confident)

^7^The combined confidence score is equal to the sum total of all other confidence scores (scores 20 or> = confident)
