# Supplementary material for: Jamie's Ministry of Food: Quasi-Experimental Evaluation of Immediate and Sustained Impacts of a Cooking Skills Program in Australia
Source: PLoS One. 2014 Dec 16;9(12):e114673. doi: 10.1371/journal.pone.0114673 (PMC4267737; doi:10.1371/journal.pone.0114673)
Supplement: S2 Table — Cooking and eating measures at baseline and follow up adjusted by age gender and levels of employment independently and all together. (DOCX) [file pone.0114673.s002.docx]

| **TABLE S2: Cooking and eating measures at baseline and follow up¹ adjusted by age^2^ gender and levels of employment^3^ independently and all together.** | | | | | | |  |
| --- | --- | --- | --- | --- | --- | --- | --- |
|  |  |  |  |  |  |  |  |
|  | **intervention group** | | | **wait list control group** | | | **Interaction effect^5^ P value** |
| **Outcome measure** | **baseline value(T1) mean (S.E)^4^** | **follow up value(T2) mean (S.E)** | **change from baseline(T2-T1) mean (S.E) P value** | **baseline value(T1) mean (S.E)^4^** | **follow up value(T2) mean (S.E)** | **change from baseline(T2-T1) mean (S.E) P value** | ***P value*** |
| ***Cooking and eating measures*** |  |  |  |  |  |  |  |
| **Cooking the main meal from basic ingredients^6^** |  |  |  |  |  |  |  |
| Age | 4.05(0.08) | 4.67(0.09) | 0.63(0.09) P<0.001 | 4.21(0.15) | 4.23(0.15) | 0.02(0.15) P =0.88 | P<0.001 |
| Gender | 4.08(0.08) | 4.72(0.09) | 0.63(0.09) P<0.001 | 4.11(0.14) | 4.11(0.15) | 0.00(0.15) P=0.990 | P<0.001 |
| Employment | 4.07(0.08) | 4.68(0.10) | 0.61(0.09) P<0.001 | 4.17(0.14) | 4.17(0.13) | 0.00(0.15) P=0.995 | P=0.001 |
| Age, Gender, Employment | 4.11(0.08) | 4.71(0.10) | 0.60(0.09) P<0.001 | 4.13(0.14) | 4.15(0.16) | 0.016(0.15) P=0.919 | P=0.001 |
| Consumption of ready- made meals at home^6^ |  |  |  |  |  |  |  |
| Age | 1.06(0.05) | 0.95(0.06) | -0.10 (0.06) P=0.100 | 1.05(0.8) | 1.19(0.10) | 0.14(0.10) P=0.18 | P=0.045 |
| Gender | 1.06(0.05) | 0.93(0.06) | -0.12(0.06) P=0.045 | 1.09(0.08) | 1.17 (0.10) | 0.08(0.10) P=0.41 | P=0.08 |
| Employment | 1.04(0.05) | 0.94(0.06) | -0.10(0.06)P=0.094 | 1.09(0.08) | 1.20(0.10) | 0.11(0.10) P=0.265 | P=0.067 |
| Age, Gender, Employment | 1.04(0.05) | 0.95(0.06) | -0.09(0.06) P=1.38 | 1.06(0.08) | 1.17(0.10) | 0.11(0.10) P=0.285 | P=0.092 |
| **Consumption of vegetables with the main meal^6^** |  |  |  |  |  |  |  |
| Age | 4.67(0.07) | 5.06(0.08) | 0.39(0.08) P<0.001 | 4.86(0.07) | 4.82(0.14) | -004(0.14) P=0.753 | P=0.007 |
| Gender | 4.67(0.07) | 5.07(0.08) | 0.40(0.08) P<0.001 | 4.83(0.13) | 4.81(0.13) | -0.02(0.14) P=0.862 | P=0.008 |
| Employment | 4.69(0.07) | 5.07(0.09) | 0.38(0.09) P<0.001 | 4.84(0.12) | 4.80(0.14) | -0.04(0.14) P=0.763 | P =0.010 |
| Age, Gender, Employment | 4.70(0.07) | 5.07(0.09) | 0.38(0.09) P<0.001 | 4.86(0.12) | 4.82(0.15) | -0.03(0.14) P=0.81 | P=0.014 |
| **Daily vegetable consumption (serves per day)** |  |  |  |  |  |  |  |
| Age | 2.46(0.05) | 2.98(0.06) | 0.52(0.06) P<0.001 | 2.54(0.09) | 2.61(0.10) | 0.06(0.10) P=0.511 | P<0.001 |
| Gender | 2.46(0.05) | 2.99(0.06) | 0.52(0.06) P<0.001 | 2.48(0.05) | 2.57(0.10) | 0.09(0.09) P=0.360 | P<0.001 |
| Employment | 2.48(0.05) | 2.98(0.06) | 0.50(0.06)P<0.001 | 2.50(0.09) | 2.59(0.10) | 0.08(0.09) P=0.384 | P<0.001 |
| Age, Gender, Employment | 2.49(0.05) | 3.00(0.06) | 0.50(0.60) P<0.001 | 2.51(0.09) | 2.58(0.10) | 0.07(0.10) P=0.71 | P<0.001 |
| **Daily fruit consumption (serves per day)** |  |  |  |  |  |  |  |
| Age | 1.64(0.04) | 1.91(0.05) | 0.27(0.05) P<0.001 | 1.65(0.07) | 1.75(0.09) | 0.10(0.08) P=0.244 | P=0.068 |
| Gender | 1.64(0.04) | 1.92(0.05) | 0.28(0.05)P<0.001 | 1.61(0.07) | 1.72(0.09) | 0.10(0.08) P=0.21 | P=0.057 |
| Employment | 1.64(0.04) | 1.91(0.05) | 0.27(0.05) P<0.001 | 1.63(0.07) | 1.72(0.08) | 0.09(0.07) P=0.234 | P=0.060 |
| Age, Gender, Employment | 1.64(0.04) | 1.91(0.05) | 0.27(0.05) P<0.001 | 1.65(0.07) | 1.74(0.08) | 0.09(0.08) P=0.250 | P=0.062 |
| **Take-away consumption^6^** |  |  |  |  |  |  |  |
| Age | 0.98(0.04) | 0.74(0.03) | -0.23(0.04) P<0.001 | 0.87(0.06) | 0.92(0.06) | 0.04(0.06) P=0.482 | P<0.001 |
| Gender | 0.98(0.04) | 0.73(0.04) | -0.25(0.03) P<0.001 | 0.94(0.07) | 0.95(0.06) | 0.01(0.06) P=0.795 | P<0.001 |
| Employment | 0.96(0.03) | 0.76(0.04) | -0.21(0.04) P<0.001 | 0.90(0.06) | 0.92(0.07) | 0.02(0.06) P=0.685 | P=0.001 |
| Age, Gender, Employment | 0.96(0.03) | 0.75(0.04) | -0.20(0.04) P<0.001 | 0.88(0.06) | 0.93(0.07) | 0.04(0.06) P=0.471 | P=0.001 |
|  |  |  |  |  |  |  |  |

¹Outcomes within each group and over time were determined by a mixed linear model for repeated measures using all available data at each time point. All means and standard errors (S.E) rounded to 2 decimal points

^2^ Age dichotomised at 50 years (below 50 years and 50 years and above)

^3^ Levels of employment where 1= full time, 2= part time, 3= home duties/carer, 4= not working (permanently ill/unemployed), 5= student, 6= retired, 7=other

^4^Baseline values were not significantly different between groups (independent t tests)

^5^A significant group x time interaction effect denotes that the response over time differed between groups

^6^ Times per week
