# Supplementary material for: Jamie's Ministry of Food: Quasi-Experimental Evaluation of Immediate and Sustained Impacts of a Cooking Skills Program in Australia
Source: PLoS One. 2014 Dec 16;9(12):e114673. doi: 10.1371/journal.pone.0114673 (PMC4267737; doi:10.1371/journal.pone.0114673)
Supplement: S3 Table — Results of ordinal logistic regression for confidence to cook questions. (DOCX) [file pone.0114673.s003.docx]

| **Table S3 : Results of ordinal logistic regression for confidence to cook questions** | | | | |
| --- | --- | --- | --- | --- |
|  |  |  |  |  |
| **Cooking confidence** | **OLR odds ratio (robust SE)** | **Confidence intervals** | **Z score** | **P value** |
| Confidence to cook from basic ingredients^3^ | 3.73 (0.59) | 2.74 -5.08 | 8.37 | p<0.001 |
| Confidence to follow a simple recipe^3^ | 3.21 (0.55) | 2.29 – 4.51 | 6.76 | p<0.001 |
| Confidence in preparing and cooking new foods and recipes^3^ | 3.61(0.6) | 2.60 – 5.01 | 7.67 | p<0.001 |
| Confidence that what one cooks will turn out well^3^ | 3.9 (0.65) | 2.82 – 5.41 | 8.19 | p<0.001 |
| Confidence to taste new foods never eaten before^3^ | 2.16 (0.35) | 1.57 – 2.95 | 4.79 | p<0.001 |

OLR = Ordinal Logistic Regression. SE = Standard error. Odds ratios refer to the group by time interaction Results obtained using Ologit in STATA 12
