# Supplementary material for: Jamie's Ministry of Food: Quasi-Experimental Evaluation of Immediate and Sustained Impacts of a Cooking Skills Program in Australia
Source: PLoS One. 2014 Dec 16;9(12):e114673. doi: 10.1371/journal.pone.0114673 (PMC4267737; doi:10.1371/journal.pone.0114673)
Supplement: S4 Table — Results of the logistic models for dichotomised confidence questions (“not confident” and “confident”). (DOCX) [file pone.0114673.s004.docx]

| **Table S4 : Results of the logistic models for dichotomised confidence questions (“not confident” and “confident”)** | | | | |
| --- | --- | --- | --- | --- |
|  |  |  |  |  |
| **Cooking confidence** | **Odds ratio (SE)** | **Confidence intervals** | **Z score** | **P value** |
| Confidence to cook from basic ingredients^5^ | 3.75 (0.84) | 2.41 – 5.81 | 5.9 | P<0.001 |
| Confidence to follow a simple recipe^5^ | 3.66 (0.92) | 2.24 – 5.98 | 5.18 | P<0.001 |
| Confidence in preparing and cooking new foods and recipes^5^ | 3.56 (0.74) | 2.37 – 5.36 | 6.09 | P<0.001 |
| Confidence that what one cooks will turn out well^5^ | 4.31 (0.89) | 2.87 – 6.46 | 7.06 | P<0.001 |
| Confidence to taste new foods never eaten before^5^ | 2.21 (0.44) | 1.50 – 3.27 | 3.99 | P<0.001 |

SE= Standard Error, Odds ratios refer to the group by time interaction. Results obtained using Xtgee in STATA 12.
